# Long non‐coding RNA Malat1 promotes neurite outgrowth through activation of ERK/MAPK signalling pathway in N2a cells

**DOI:** 10.1111/jcmm.12904

**Published:** 2016-07-04

**Authors:** Lei Chen, Peimin Feng, Xi Zhu, Shixu He, Jialan Duan, Dong Zhou

**Affiliations:** ^1^ Department of NeurologyWest China Hospital of Sichuan UniversityChengduChina; ^2^ Department of GastroenterologyAffiliated Hospital of Chengdu University of Traditional Chinese MedicineChengduChina

**Keywords:** LncRNA, Malat1, neurite outgrowth, ERK

## Abstract

Accumulating evidence suggests that long non‐coding RNAs (lncRNAs) are playing critical roles in neurogenesis, yet the underlying molecular mechanisms remain largely elusive. Neurite outgrowth is an early step in neuronal differentiation and regeneration. Using *in vitro* differentiation of neuroblastoma‐derived Neuro‐2a (N2a) cell as a model, we performed expression profiling to identify lncRNAs putatively relevant for neurite outgrowth. We identified that Metastasis‐associated lung adenocarcinoma transcript 1 (Malat1) was one of the most significantly up‐regulated lncRNAs during N2a cell differentiation. Malat1 knockdown resulted in defects in neurite outgrowth as well as enhanced cell death. To pinpoint signalling pathways perturbed by Malat1 depletion, we then performed a reporter‐based screening to examine the activities of 50 signalling pathways in Malat1 knockdown cells. We found that Malat1 knockdown resulted in conspicuous inhibition of Mitogen‐Activated Protein Kinase (MAPK) signaling pathway as well as abnormal activation of Peroxisome proliferator‐activated receptor (PPAR) and P53 signalling pathway. Inhibition of ERK/MAPK pathway with PD98059 potently blocked N2a cell neurite outgrowth, whereas phorbol 12‐myristate 13‐acetate‐induced ERK activation rescued defects in neurite outgrowth and cell death induced by Malat1 depletion. Together, our results established a critical role of Malat1 in the early step of neuronal differentiation through activating ERK/MAPK signalling pathway.

## Introduction

Recent advances in high‐throughput sequencing technology and genome‐wide transcriptional analysis have allowed the identification of a large number of transcripts with no protein‐coding potential. It is now well known that a large proportion of the genomes of higher vertebrates is transcribed into various non‐coding RNAs (ncRNAs), including ribosomal RNAs, transfer RNAs (tRNAs), small interfering RNAs (siRNAs), microRNAs (miRNAs), PIWI‐interacting RNAs (piRNAs) as well as long ncRNAs (lncRNAs) 1, 2. LncRNAs are currently defined as ncRNAs longer than 200 nucleotides in length 3, 4. They are expressed in a tissue‐specific manner and are dynamically regulated during development. Similar to protein‐coding genes, most lncRNAs are transcribed by RNA polymerase II, and are spliced and polyadenylated. Although initially thought to be transcriptional noise *per se*, lncRNAs are now considered functional RNAs with divergent roles in transcriptional and post‐transcriptional regulation of gene expression. For example, through interaction with chromatin‐modifying complexes and/or transcription machinery, lncRNAs could function as critical regulators of transcription activity and mRNA expression of target genes 5, 6. In addition, some lncRNA might act as competing endogenous RNAs (ceRNAs) that compete with protein‐coding genes for the binding to miRNAs and other regulatory factors 7. It has also been shown that lncRNA might serve as precursors to small RNAs and regulators of mRNA degradation 8. Attesting to their important biological functions, aberrant expression of lncRNAs have been implicated in various human diseases such as cancer 9, whereas some lncRNAs are clearly subject to considerable purifying selection and are well conserved across different species 10.

Recently, lncRNA has emerged as a novel regulator for neurogenesis 11. For example, depletion of lncRNA Dali disrupted expression of transcription factor Pou3f3 and several other key regulators of neural differentiation, resulting in compromised neurite outgrowth 12. Mouse model with linc‐Brn1b knockout exhibits dramatic abnormalities in the generation of upper‐layer II–IV neurons in the neocortex 13. Moreover, depletion of LncRNA BC1 results in abnormal activation of neuronal excitability 14 and is implicated in the mouse models of epilepsy. It has also been shown that lncRNA RMST could activate transcription factor SOX2 to promote the expression of several genes essential for neurogenesis. Loss of RMST resulted in compromised differentiation of neurons in culture along with overproduction of glial markers in these cells 15. It has also been shown that uc.217 regulates neurite outgrowth in dorsal root ganglion neurons 16. These results are consistent with lncRNAs being critical regulators of neuronal differentiation, development and regeneration.

Neurite outgrowth is a fundamental process of neuronal differentiation during early neuronal development. It also plays a critical role in neuronal regeneration and injury response. Neurite outgrowth is a biological event involving complex regulations on gene expression and signal transduction 17, 18. Although a few lncRNAs have been identified to be essential for neurite outgrowth, the function of the vast majority of neuronal‐expressed lncRNAs remains largely unknown. In this study, we aimed to systematically investigate the changes in lncRNA expression during neurite outgrowth, and to determine the putative mechanisms underlying the regulatory functions of the lncRNAs. To this end, using a well‐established model of mouse neuroblastoma N2a cell neurite outgrowth by serum withdrawal and retinoic acid (RA) treatment 19, we determined changes in expression of 90 highly conserved lncRNAs during differentiation using LncProfiler qPCR array. This allowed us to identify lncRNA Malat1 as one of the most significantly induced lncRNA associated with neurite outgrowth. In line with this, our data supported that Malat1 played a critical role in promoting neurite outgrowth. We then further investigated the role of Malat1 in signalling transduction using a reporter‐based screen and functional assays.

## Materials and Methods

### N2a cell culture and differentiation

N2a cells obtained from ATCC were cultured in DMEM (Thermo Fisher, Waltham, MA, USA) containing 10% heat‐inactivated FCS (Gibco, Carlsbad, CA, USA) and penicillin/streptomycin (Gibco), at 37 degree with 5% CO_2_. For neurite outgrowth assay, cells were seeded in 24‐well culture plate at an initial density of 5000 cells per well containing complete growth medium (1 ml/well) for 18 hrs. To induce cell differentiation, the complete medium was replaced by DMEM with 0.1% serum medium and 5 μM RA and continued culturing for 24 hrs. MAPK inhibitor PD98059 and activator phorbol 12‐myristate 13‐acetate (PMA) were purchased from Cell Signaling, Beverly, MA, USA.

### Measurement of Neurite Outgrowth

After staining, cells were viewed using an inverted microscope (Eclipse Ti‐E Inverted Microscope; Nikon, Tokyo, Japan). Neurite length in each cell was defined as the distance between the centre of the cell soma and the tip of its longest neurite. Only neurite emerging from an isolated cell, but not from a clump of cells, was considered, and it should not contact other cells or neurites. For each group, the lengths of neurite from 100 randomly selected cells were measured with ImageJ software and the mean ± S.E.M. values were calculated.

### LncRNA expression profiling

Following different treatment, total RNA from N2a cells was extracted using Trizol reagent (Invitrogen, Carlsbad, CA, USA). LncRNA expression profiling was performed with SBI's LncProfiler qPCR array according to the instructions of the manufacturers (System Biosciences, Palo Alto, CA, USA). This assay sets allow us to simultaneously quantitate 90 lncRNAs, five housekeeping reference controls and one negative control. Real‐time PCR analysis was performed with a CFX Connect Real‐Time PCR Detection System (Bio‐Rad, Hercules, CA, USA). Experiments were performed in triplicates and the values were calculated as mean ± S.E.M.

### Signal transduction reporter array

Cignal Signal Transduction Reporter Array (Qiagen, Valencia, CA, USA) was used to simultaneously investigate alternations in the activities of 50 canonical signalling pathways in response to Malat1 depletion. Cells were transfected with antisense oligonucleotides‐targeting Malat1 for 24 hrs and were subsequently transfected with a mixture of a transcription factor‐responsive firefly luciferase reporter and a constitutively expressing Renilla construct. The relative activity of each pathway was decided by luciferase/Renilla and normalized by untreated controls. Experiments were performed in triplicates and the values were calculated as mean ± S.E.M.

### Western blot and immunofluorescence

For Western blot, 10 μg proteins were separated on SDS–PAGE and subjected to immunoblotting using AKT (1:1000; Cell Signaling, Beverly, MA, USA), p‐AKT (1:1000; Cell Signaling, Beverly, MA, USA), c‐Jun N‐terminal kinase (JNK; 1:1000; Cell Signaling, Beverly, MA, USA), p‐JNK (1:1000; Proteintech, Rosemont, IL, USA), extracellular signal‐regulated kinase (ERK; 1:1000; Abcam, Cambridge, UK), p‐ERK (1:1000; Cell Signaling, Beverly, MA, USA), P38 (1:2000; Cell Signaling, Beverly, MA, USA), p‐P38 (1:2000; Cell Signaling beverly, MA, USA) and Tubulin (1:5000; Sigma‐Aldrich, Natick, MA, USA) antibodies. For immunofluorescence, after fixation with 4% paraformaldehyde, permeabilization with 0.1% Triton and blocking with 5% BSA in PBS, N2a cells were incubated with anti‐Tubulin (1:1000; Sigma‐Aldrich) and Cy3 sheep antimouse IgG (1:1000; Sigma‐Aldrich) was used as secondary antibody. Hoechst 33342 was used as a stain for nuclei.

### Malat1 knockdown

Phosphorothioate internucleosidic linkage‐modified DNA antisense oligonucleotides were used to knockdown MALAT1 in N2a cells or as a scrambled control. Oligos sequences were as follows (Shanghai Shengong, Shanghai, China): siMalat1: 5′‐AGGCAAACGAAACATTGGCA‐3′; Scr: 5′‐GCTTAGGCAGTGCCAGAATT‐3′. The oligonucleotides were transfected to cells for 24 hrs at a final concentration of 100 nM using Lipofectamine RNAimax reagent (Invitrogen).

### Flow cytometry

N2a cells were seeded in 6‐well plates for 24 hrs and then transfected with siMalat1 or control oligonucleotides. Following different treatment, cells were trypsinized (Invitrogen), washed twice with PBS and resuspended in Annexin V binding buffer (Invitrogen). They were then stained with Annexin V/PI staining kit (Invitrogen) for 15 min. in the dark, and the cell populations were analysed by a FACSCalibur Flow Cytometer (BD, Franklin Lakes, NJ, USA). A total of 3000 cells were counted in each assay.

## Results

### Alternations in lncRNA expression during neurite outgrowth

**Figure 1:**
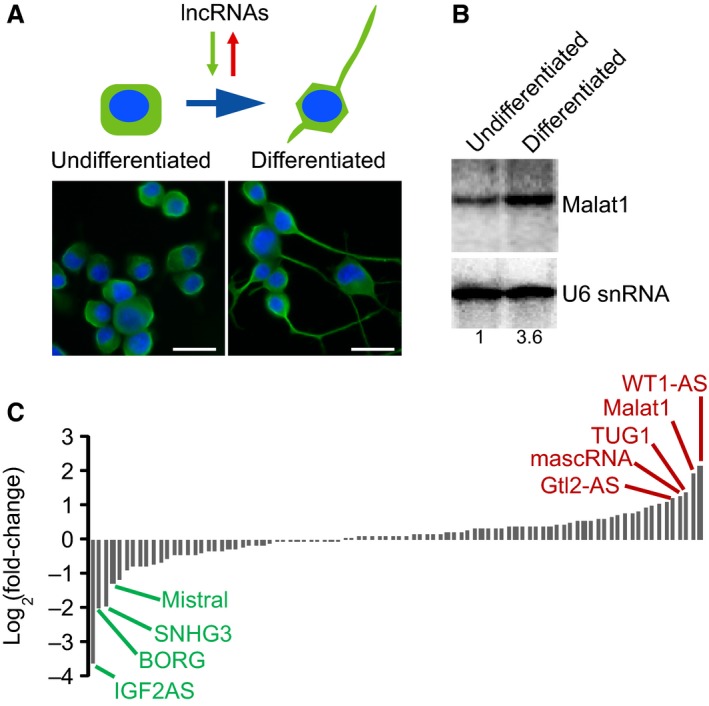
Alternations in lncRNA expression during N2a cell differentiation. (**A**) Undifferentiated and differentiated N2a cells stained with Tubulin (Green) and nucleus (blue), scale bar: 50 μm. (**B**) Changes in expression of 90 lncRNAs during N2a cell differentiation. Fold change was log2 transformed and the data present the mean value from three biological replicates. The histogram shows the fold changes in LncRNAs which were sorted by fold changes. The details for fold changes and corrected *P*‐value for each lncRNA expression was listed in Table S1. (**C**) Northern blot analysis of Malat1 transcripts in undifferentiated and differentiated N2a cells.

Differentiation of mouse neuroblastoma N2a cell by serum withdrawal and RA treatment presents a well‐established model of neurite outgrowth *in vitro*. As shown in Figure 1A, after treatment for 24 hrs, we observed robust neurite formation and outgrowth in differentiated N2a cells as compared with undifferentiated cells. This robust *in vitro* model thus allowed us to investigate the alternations in lncRNA expression associated with N2a differentiation.

To this end, we measured expression levels of 90 conserved lncRNAs in N2a cells before and after differentiation using LncProfiler qPCR Arrays (Table S1). Expression level of each lncRNA was measured with triplicates in differentiated and undifferentiated N2a cells using this high‐throughput real‐time PCR array, and the difference in lncRNA expression was determined using Student's *t*‐test with Bonferroni corrections for multiple comparisons. This allowed us to identify several lncRNAs that are significantly altered during N2a differentiation. As shown in Figure 1B, lncRNAs with robustly enhanced expression (>2‐fold, corrected *P* < 0.05) included WT1‐AS, Malat1, TUG1, mascRNA and Gtl2‐AS, whereas significantly down‐regulated (>2‐fold, corrected *P* < 0.05) lncRNAs included IGF2AS, SNHG3, BORG and Mistral (Table S1). We have paid particular attention to Malat1, which has been implicated in neuronal cell development and in neurodegenerative diseases 20, 21. Northern blot analyses validated the results of real‐time PCR that Malat1 expression was enhanced by about 3.6‐fold in differentiated N2a cells (Fig. 1C).

### Elevated expression of Malat1‐enhanced neurite outgrowth

**Figure 2:**
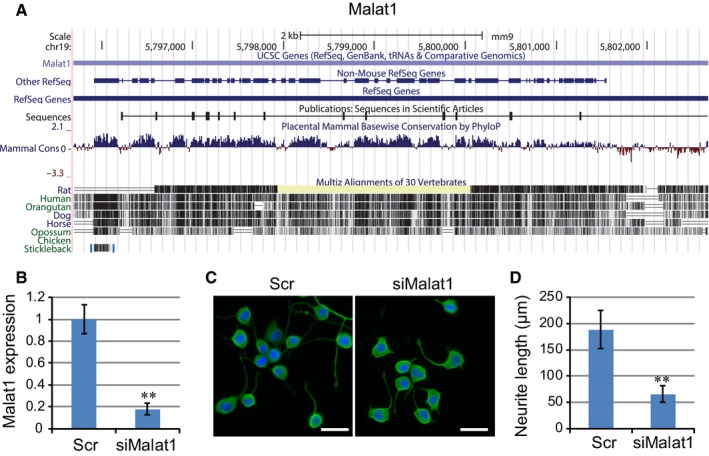
Malat1 is a conserved lncRNA that promotes neurite outgrowth. (**A**) Graphical views showing multi‐species comparisons of Malat1 using UCSC genome browser. The conservation scores were indicated by the blue and red peaks. (**B**) Changes in Malat1 expression following treatment with antisense oligo targeting Malat1 or scrambled control using real‐time PCR. Data show the mean ± S.E.M. values of three replicates (**P < 0.001). (**C**) Neurite outgrowth in cells transfected with siMalat1 or scrambled oligos. (**D**) Quantification of neurite outgrowth in cells transfected with siMalat1 or scrambled oligos. The lengths of neurite from 100 randomly selected cells were measured with ImageJ software and the mean ± S.E.M. values were calculated (**P < 0.001).

One of the most significantly induced lncRNA during N2a differentiation was Malat1, which was initially characterized as a long polyadenylated ncRNA overexpressed in cancer 22, 23. Phylogenetic analysis using the UCSC Genome Browser (http://genome.ucsc.edu/) revealed that Malat1 is highly conserved among mammals, with a 90% similarity between human and mouse, suggesting important function of the lncRNA (Fig. 2A). Interestingly, expression profiling of Malat1 in different human tissues by lncRNAdb database 24 revealed that Malat1 is highly expressed in brain (Fig. S1), suggesting its implications in neuron biogenesis and function. Previous studies suggested that Malat1 is localized to nuclear speckles, which contain a large number of nuclear proteins involved in mRNA splicing and transport, and might function in epigenetic and transcriptional regulation of gene expression.

The highly restricted evolutionary conservation among different species as well as enhanced expression of Malat1 during N2a cell differentiation suggested that it may play an important role in neurite outgrowth. We therefore depleted Malat1 expression in N2a cells using antisense oligonucleotides (siMalat1) to address the potential role of Malat1. As shown in Figure 2B, real‐time PCR results suggested that Malat1 knockdown resulted in over fivefold decrease in Malat1 level as compared with control knockdown using a scrambled oligo (*P* < 0.001, Student's *t*‐test). Following Malat1 knockdown, N2a cell differentiation was induced using RA in combination with serum withdrawal. Interestingly, Malat1 knockdown led to a dramatic decrease in the average neurite length per cell, indicating a potent repression of neurite outgrowth (Fig. 2C and D, in μm; Scr, 188.62 ± 36.21; siMalat1, 65.63 ± 15.35; *P* < 0.001, Student's *t*‐test). Together, these results supported a critical role of Malat1 in promoting neurite outgrowth in N2a cells.

### Malat1 knockdown resulted in inhibition of ERK/MAPK signalling pathway

**Figure 3:**
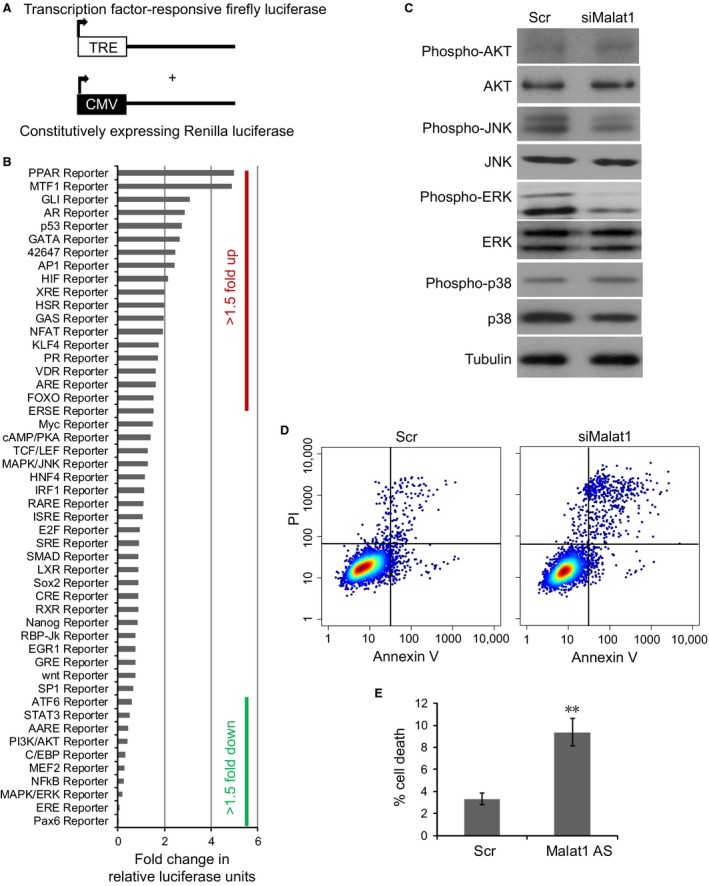
Alternations in the activities of signalling pathways upon Malat1 depletion. (**A**) Schematics of the transcription factor‐responsive firefly luciferase reporter and the constitutively expressing Renilla construct. (**B**) Histogram shows the fold changes for the activities of different signalling pathways, as indicated by reporter activity. (**C**) Western blot analysis of the expression levels and phosphorylation levels of different MAPK components. (**D**) Flow cytometry analysis of cell death in cells transfected with scramble or siMalat1 oligo. Cells were stained with Annexin V and PI. (**E**) The percentage of dead cells in (**D**), which could be stained with Annexin V. Data show the mean ± S.E.M. values of three replicates (**P < 0.001).

To investigate the molecular mechanisms underlying how Malat1 contributes to neurite outgrowth, we used Cignal Signal Transduction Reporter Array to simultaneously investigate the activities of 50 canonical signalling pathways upon Malat1 depletion in undifferentiated N2a cells. This assay involved a mixture of a pathway‐specific transcription factor‐responsive firefly luciferase reporter, which contains a specific transcription factor‐responsive element in the promoter (TRE), and a constitutively expressed Renilla luciferase reporter, which were co‐transfected to monitor alternations in the activity of that signalling pathway (Fig. 3A).

This high‐throughput dual‐luciferase assay allowed us to identify several relevant pathways putatively affected by Malat1 knockdown (Table S2). As shown in Figure 3B, our result identified several signalling pathways that were significantly repressed upon Malat1 depletion (fold change >2, corrected *P* < 0.05), including Pax6, ERE, MAPK/ERK, NFkB, MEF2, C/EBP, PI3K/AKT and AARE signalling pathways. We also identified several signalling pathways that were significantly induced by Malat1 depletion (fold change >2, corrected *P* < 0.05), including PPAR, MTF1, GLI, AR, p53, GATA, AP1 and HIF signalling.

Of note, we identified MAPK/ERK signalling as one of the most significantly repressed pathways upon Malat1 depletion. There are three major subfamilies of MAPKs, namely, ERKs, JNKs and p38‐MAPKs, that are involved in a wide range of biological functions such as development, stress response and apoptosis. Previous results also showed that serum deprivation‐induced N2a cell differentiation is associated with activation of ERKs and AKT signalling. We thus focused on MAPK signalling pathways and AKT pathways and used Western blot to investigate alternations in the activities of these pathways upon Malat1 depletion in differentiated N2a cells. Our results suggested that there was no obvious activation of AKT signalling, indicated by AKT phosphorylation, in either scramble knockdown or Malat1 knockdown cells. Interestingly, the level of ERK phosphorylation was significantly reduced in Malat1 knockdown cells, whereas JNK or p38 phosphorylation was not significantly altered. Consistent with previous result, we showed that treatment with MAPK/ERK inhibitor PD98059 potently abolished neurite outgrowth in N2a cells, supporting the role of MAPK/ERK signalling in promoting N2a differentiation (Fig. S2) 25. These data suggested that Malat1 might mediate neurite outgrowth in N2a cells through activating MAPK/ERK signalling pathway.

Moreover, it has been shown that MAPK/ERK signalling is important for cell survival, suggesting enhanced cell death in Malat1 knockdown cells. This is in line with abnormal activation of PPAR and P53 signalling upon Malat1 depletion, which might lead to apoptotic cell death induction. To test this hypothesis, N2a cells with control knockdown or Malat1 knockdown were stained with PI/Annexin V followed by flow cytometry analysis. As shown in Figure 3D and E, the rate of apoptosis in Malat1 knockdown cells was significantly higher than in control knockdown cells (9.36 ± 1.25% *versus* 3.32 ± 0.52%, **P < 0.001, Student's *t*‐test), consistent with Malat1 being important in facilitating cell survival.

### ERK activation rescued defects in neurite outgrowth induced by Malat1 depletion

**Figure 4:**
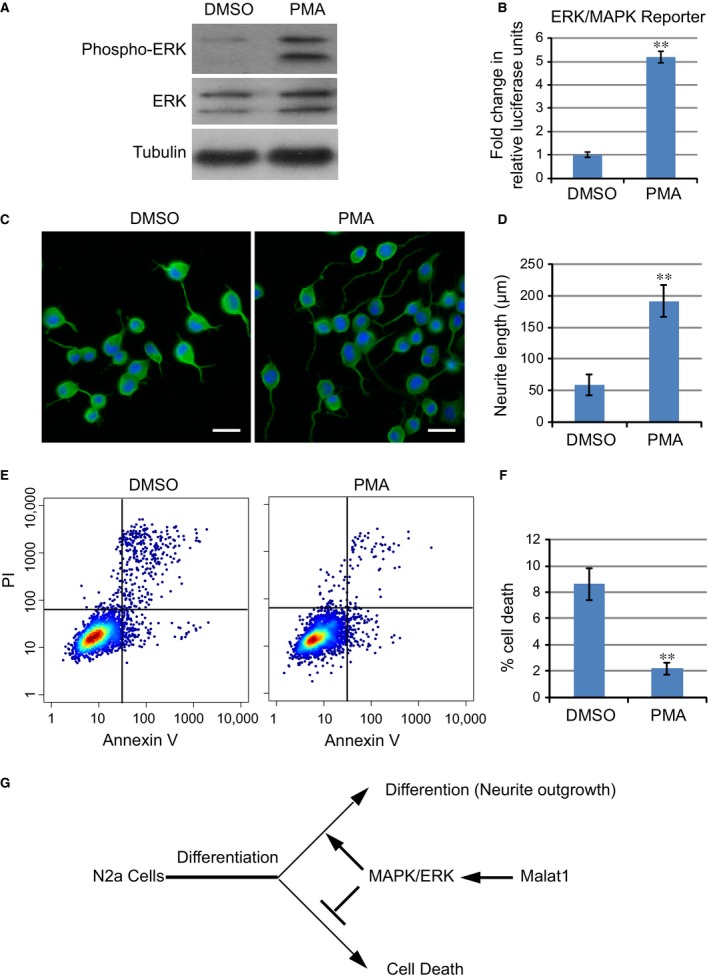
Activation of ERK/MAPK signalling rescued defects caused by Malat1. (**A**) Western blot of ERK and ERK phosphorylation in cells transfected with siMalat1. (**B**) Activities of the luciferase reporter responsible for ERK/MAPK activity. Data show the mean ± S.E.M. values of three replicates (**P < 0.001). (**C**) Neurite outgrowth in Malat1 knockdown cells treated with DMSO or PMA. (**D**) The lengths of neurite from 100 randomly selected cells and the mean ± S.E.M. values were calculated (**P < 0.001). (**E**) Flow cytometry analysis of cell death in Malat1 knockdown cells transfected with DMSO or PMA. (**F**) Mean ± S.E.M. values of three replicates for (**D**) (**P < 0.001). (**G**) A model regarding how Malat1 regulates neurite outgrowth and inhibits cell death.

We then asked whether the potential modulatory effects of Malat1 on neurite outgrowth and cell survival were dependent on MAPK/ERK signalling. If so, forced activation of ERK should be able to rescue the defects caused by Malat1 knockdown. Phorbol 12‐myristate 13‐acetate is a well‐established activator of PKC, which is a key regulator of MAPK/ERK signalling pathway 26. We thus examined the effects of PMA on neurite outgrowth of N2a cells lacking Malat1. As expected, Malat1 knockdown cells treated with 250 nM PMA showed a potent increase in ERK phosphorylation, whereas the total ERK protein expression level remained unaffected (Fig. 4A). Consistently, activity of luciferase reporter responsive to MAPK/ERK signalling was enhanced by more than fivefold in cells treated with PMA as compared with cells without PMA treatment (Fig. 4B), indicating forced activation of MAPK/ERK pathway by PMA in these cells.

Interestingly, our results showed that addition of PMA potentiated the neurite outgrowth process and largely rescued the defects caused by Malat1 depletion. As shown in Figure 4C and D, Malat1 knockdown cells treated with PMA showed significantly higher average neurite length as compared with cells treated with DMSO (in μm; DMSO, 58.57 ± 16.71; siMalat1, 191.62 ± 25.35; *P* < 0.001, Student's *t*‐test). Moreover, the percentage of cell death was likewise decreased significantly as a result of PMA treatment (Fig. 4E and F, 8.66 ± 1.45% *versus* 2.12 ± 0.61%, **P < 0.001, Student's *t*‐test). Together, these data suggested a model that Malat1 played a critical role in regulating the transition of N2a cells from an undifferentiated to a differentiated status by inducing neurite outgrowth and inhibiting cell death. Although the underlying mechanisms remained unknown, these results provided robust evidence that Malat1 function in N2a cell differentiation was dependent on MAPK/ERK signalling (Fig. 4G).

## Discussion

In this study, we identified Malat1 as one of the most significantly up‐regulated lncRNAs during N2a cell differentiation. We also provided robust evidence that Malat1 played an important role in stimulating neurite outgrowth and survival during N2a cell differentiation. Furthermore, we identified several signalling pathways possibly disrupted by Malat1 depletion with an unbiased pathway activity screen. We argued that the activity of MAPK/ERK signalling pathway regulated by Malat1 was critical in N2a cell differentiation. Chemical inhibition of MAPK/ERK signalling resulted in potently impaired neurite outgrowth, whereas forced activation of MAPK/ERK signalling by PMA could largely rescue the defects caused by Malat1 depletion. Together, these results supported a novel role of Malat1 in promoting neurite outgrowth and survival during N2a cell differentiation, probably through regulating MAPK/ERK signalling pathway, although the underlying mechanisms remain to be elucidated.

Accumulating evidence suggests that lncRNAs are temporally and spatially expressed, and their functions are diverse and complicated 1, 3, 27. The mechanisms might involve transcriptional and post‐transcriptional regulation of gene expression, and/or interaction with critical RNA/protein molecules. Recent studies have linked lncRNAs to the regulation of a wide range of biological functions, and the disruption of some of these functions, such as genomic imprinting and gene expression, are immediately relative to various human diseases such as cancer 9, 10, 11. It is well known that Malat1 is highly expressed in several cancers and overexpression of MALAT1 facilitates cancer cell proliferation and tumour metastasis. It has been shown that MALAT1 is localized to nuclear speckles and could regulate the splicing of subset of pre‐mRNAs by interaction with SR proteins and splicing factors 28. Specifically, Malat1 might function as a molecular sponge to titrate the local concentration of RNA binding proteins and creates a gradient of functionally competent SR proteins and splicing factors in the gene locus to control alternative splicing 28. During the transition from undifferentiated status to differentiated status, a highly programmed alternation in gene expression might occur and a subset of key regulators, such as transcription factors and protein kinases, might need to be transcribed, spliced and activated in a timely manner to ensure efficient differentiation. Depletion of Malat1 might disrupt this tightly controlled gene expression network and thus led to defects in signal transduction and neurite outgrowth. Moreover, loss of Malat1 might also disrupt the architecture of nuclear speckles and other nuclear organization that are essential for the differentiation of neurons and other cell types. Consistent with our results, several previous studies have supported the critical role of Malat1 in neuronal cells. For example, it has been shown that Malat1 regulates synaptogenesis by modulating global gene expression 20. The roles of Malat1 in brain development, functional diversification, stress response and neurodegenerative diseases have also been widely reported 21, 29.

Of note, our results showed that PMA‐induced ERK phosphorylation could largely rescue the defects in neurite outgrowth in Malat1 knockdown cells, supporting that the function of Malat1 was at least partly dependent on ERK/MAPK signalling pathway. Consistent with this notion, it has been shown that MALAT1 depletion in lung cancer cells could significantly reduce the expression levels of Glypican 6 and C‐X‐C motif chemokine 5 to block signalling transduction upstream of ERKs 22. As these upstream regulators responsive to external signals, such as G‐protein coupled receptors and growth factor receptors, are likely expressed in a tissue‐specific manner and would rapidly change their expression during differentiation and development, loss of Malat1 might have a potent influence on their transcription and splicing, and thus led to inhibition of signal transduction of the pathway.

Interestingly, we observed that disrupted neurite outgrowth was accompanied by enhanced cell death in Malat1 knockdown cells. This suggests that neural development is a complex process that integrates proliferation, differentiation and programmed cell death or autophagy. Our findings that depletion of MALAT1 leads to activation of p53 are consistent with several previous studies 23, 30 and might contribute to cell death in Malat1 knockout cells. In response to stress stimuli such as serum withdrawal or RA treatment, cells might face critical decision making in terms of differentiation or cell death during the transition, presumably determined by different external signals and/or internal signal transduction whose nature yet remains to be elucidated. Moreover, we acknowledge that there are several limitations to this study, for example, N2a cell is a cancer‐derived cell line which might not be the best model to study neurological processes, and our targeted approaches might lack the power to detect alternations of several important lncRNAs and pathways which were otherwise not present in the arrays. Nevertheless, our results were consistent with MAPK cascades being important in cellular proliferation, differentiation, stress response and survival. Further genome‐wide proteomics studies, including large‐scale protein phosphorylation analysis, might help to directly identify the targets of Malat1 and to unveil the mechanisms underlying the role of Malat1 in promoting/maintaining the activity of MAPK cascades. As we mentioned, neurite outgrowth is only a small early step of neural development. As compared with the well‐established functions of protein‐coding genes, functions have only been unveiled for a small number of lncRNAs. Further studies are required to investigate the mechanisms by which lncRNAs might function in regulating gene expression and signalling transduction in different cell types during neuronal development, and to determine how gain and loss of function of individual lncRNAs may contribute to the pathogenesis neurodegenerative diseases and cancer.

## Conflict of interest

The authors claim no conflict of interest.

## Supporting information


**Figure S1** Expression profiling of Malat1 in different human tissues.

**Figure S2** ERK/MAPK inhibitor PD98059 impaired neurite outgrowth in N2a cells. (**A**) Differentiation of N2a Cells treated with DMSO or ERK/MAPK inhibitor PD98059. (**B**) Quantification of neurite outgrowth in cells treated with DMSO or PD98059. The lengths of neurite from 100 randomly selected cells were measured with ImageJ software and the mean ± S.E.M. values were calculated (*P < 0.05).

**Table S1** Changes in LncRNA expression during N2a cell differentiation.
**Table S2** Alternations in signalling pathway activities upon Malat1 depletion.
